# Invasive endemic fungi of the Western Hemisphere

**DOI:** 10.1080/21505594.2019.1664719

**Published:** 2019-10-02

**Authors:** Joshua Fierer

**Affiliations:** a Medical and Research Services, VA Healthcare San Diego, CA, USA; b Division of Infectious Diseases, Department of Medicine, UC San Diego School of Medicine, La Jolla, CA, USA

This Special Issue deals with five systemic pathogenic fungi, all endemic in the Western Hemisphere, four of which are thermally dimorphic and the fifth is *Cryptococcus neoformans*, an important invasive yeast that can infect apparently immunocompetent hosts as well as immunocompromised people. The prevalence of asymptomatic cryptococcal infections is not known as there is no skin test antigen available, but using skin testing to diagnose prior infection with the dimorphic fungi revealed that the vast majority of infections with dimorphic fungi are asymptomatic [1]. Nevertheless, the overall economic and human cost of symptomatic invasive fungal infections is quite large. In 2014 in the United States, fungal infections resulted in ~75,000 hospitalizations with an estimated cost of $4.6 billion, and the four pathogens featured in this symposium that occur in the United States accounted for about 23% of those hospitalizations [2]. Paracoccidioidomycosis (PCM), which is endemic in Central and South America, is not a reportable disease in the endemic countries, so we do not have reliable incidence or prevalence figures from that region. However, in Brazil hospitalization rates for PCM vary from 1.6 to 8.3/100,000 residents in different regions of the country, indicating the enormity of that problem in Brazil [3]. PCM is also endemic in the other northern countries in South America, and in Central America where the most cases are reported from Mexico, primarily from the rural southern states that have heavy rainfall and a lot of vegetation. Despite the millions of infections that are caused by thermally dimorphic fungi worldwide there are no vaccines and few if any drugs have been developed and FDA-approved to treat any of three dimorphic pathogens featured in this special issue.

The invasive dimorphic fungi in the Western Hemisphere have many commonalities, which is to be expected since they are all members of the order Onygenales. Although it was originally believed that each of the diseases caused by those fungi was caused by a single species, genome sequencing and, in some cases, actual mating experiments have revealed that each disease can be caused by two or more different species [4–7]. The diseases they cause are with rare exceptions initiated by inhalation of asexual spores (or in the case of cryptococcosis, by inhaled desiccated yeast), and person to person transmission is almost unheard-of. The inhaled spores undergo a complex transformation into yeast that multiply inside macrophages in the host (*Histoplasma, Paracoccidioides, Blastomyces*), or transform into spherules (*Coccidioides*) that are primarily found extracellular because they only are small enough to be ingested while they are immature round cells. Pathogenic cryptococci are not dimorphic, but they are also facultative intracellular pathogens that multiply inside or are extruded by macrophages [8] but can grow extra-cellularly, which is most often seen in the cerebral spinal fluid. In all cases elevated temperature is a trigger for the morphological transformation from spores to the tissue invasive forms.

The articles by Beyhan and Sil [9], McBride et al. [10], and Kirkland and Fierer [11] review what is known about the changes in gene expression that accompany the transition from spore to the pathogenic forms. The temperature-driven transformation in *H. capsulatum* is driven by four highly conserved factors, Ryp (Required for Yeast Phase)1, Ryp2, Ryn3, and Ryp4. Ryp factors belong to distinct families of proteins that control the developmental transitions in fungi. Ryp1, Ryp2, and Ryp3 are transcription factors that along with Ryp4 regulate their own and each other’s expression [9] and many of the known virulence genes in *H capsulatum*. These same transcription factors are conserved in *Blastomyces* and are required for yeast development and for the expression of multiple virulence factors [10]. The Ryp family of proteins is also present and expressed by spherules of *C. immitis* RS, but as yet their role in regulating the metamorphosis of spores into spherules is still unknown [12], and it is not yet known which of the differentially expressed genes in spherules are required for the transition from spores, the development of endospores inside spherules, or survival in a mammalian host.

These three papers also discuss the genetic regulation of virulence. Virulence of microorganisms, or their ability to cause disease of varying severity, is almost always contextual. Different hosts can vary greatly in their susceptibility to a given pathogen, and many known and unknown conditions may modify the severity of infection in members of the same species, such as prior exposure to the antigens, maternal antibodies transmitted to newborns, co-existing infections, and perhaps the composition of the host microbiome. The adaptive immune response, especially the development of pathogen-specific CD4 + T cells, is crucial for protection against these organisms, as illustrated by the increased severity of infections in people with advanced AIDS. These protective CD4 + T cells recognize fungal antigens through their T cell receptors and then secrete protective cytokines such as interferon-gamma (IFNγ), GM-CSF, and IL-17, cytokines that are important for host resistance to the dimorphic pathogens and *Cryptococcus*. The immune response may be so effective that the initial infection is sub-clinical, as usually happens with histoplasmosis and coccidioidomycosis, but after sub-clinical histoplasmosis organisms can remain viable for years, held in check, but not eliminated by the adaptive immune response, so that when that immune response is attenuated or eliminated by disease or medication, severe disease ensues even in people who have moved out of the endemic area decades before they relapse [13].

Four of the manuscripts deal in some depth with the immune response to the dimorphic fungi. Innate immune cells in the lung are the first ones to encounter inhaled spores but little is known about the responses of alveolar macrophages and lung dendritic cells to the spores or the newly minted yeast. Both of those phagocytic myeloid cells express C type lectins on their outer cell membranes that recognize specific carbohydrates that are components of fungal cell walls, and not expressed on host cells. Both *Histoplasma* and *Blastomyces* avoid recognition by the C type lectin Dectin-1 (Clec7a) by downregulating β-glucan synthesis and burying the remaining β glucan under a layer of α glucan. For *Coccidioides* and *Paracoccidioides*, Dectin-1 is the most important lectin receptor and binding by both fungi triggers a signaling cascade that leads to transcriptional activation of numerous pro-inflammatory genes and signaling cytokine genes that in mice lead to protective Th1/Th17 immune responses. Dectin-2 is important for protection against experimental blastomycosis but not coccidioidomycosis [14]. As is emphasized in the paper by Calich et al., in PCM T_reg_ cells play an important role in the control of inflammation that can otherwise result in fatal pneumonia, but they also impair the adaptive immune response needed to eliminate the yeast from tissue [15]. Some of the negative effects of T_reg_ cells is mediated by secreted IL-10. IL-10 plays a similar negative role in the immune response to *C. immitis* in mice [11]. There is evidence that people who develop chronic PCM also make a robust T_reg_ response [15], but this has not been established in human coccidioidomycosis.

All of the papers are concerned with virulence factors. The capsule of *C. neoformans* and *C. gattii* is the most important virulence factor, and the paper by Casadevall et al. [16] reviews the structure and biology of the capsule and capsule synthesis. They are remarkably large, complex polysaccharide structures (PS) that encase the yeast and prevent phagocytosis of unopsonized organisms. Either antibody or complement can be opsonic, but capsules do not generate an opsonic antibody response during infection, and complement may attach too far from the surface to be an effective opsonin. The capsule structure is not static and after synthesis, it has to be rearranged to allow daughter cells to bud off, become encapsulated, and separate from the mother cell without leaving a permanent open channel. Although there is no evidence that immunoglobulin deficiencies predispose to cryptococcal infection, Casadevall has pioneered immunotherapy with monoclonal antibodies and he here suggests that protective monoclonals cause hydrolysis of the PS and therefore small molecules that inhibit capsular synthesis may be therapeutic [16].

Both *Histoplasma* and *Blastomyces* have several known virulence factors, established by targeted gene mutations. Some virulence factors can be classified as defensive, such as superoxide dismutase, and some are scavengers of vital micro-nutrients such as iron and zinc. IFNγ-activated macrophages compete with the intra-cellular fungi for these nutrients, which is a form of nutritional immunity [17]. *Blastomyce*s and *Histoplasma* share some virulence factors, but the former has a unique protein BAD1 (Blastomyces adhesion protein 1) that not only mediates adhesion to host cells but stimulates macrophage and PMN production of TNFα, a cytokine that is important for stable granuloma formation and therefore resistance to all the dimorphic fungi. Since many *Blastomyces* antigens cross-react with *Histoplasma* antigens, and the endemic areas in the USA partially overlap, BAD1 may be useful for measuring the *Blastomyces*-specific immune response, for both diagnosis and epidemiological purposes. Much less is known about the virulence factors of *Coccidioides* as it is difficult to make targeted mutations. However, the lesions produced by this pathogen are very alkaline, mediated by a potent fungal urease and a ureidoglycolate hydrolase, and a double mutant does not increase the pH of infected tissues and is highly attenuated [18]. The ability of *Coccidioides* to form mature spherules is crucial for virulence as two mutants that cannot convert to spherules are avirulent. One avirulent strain has a double mutation that eliminates two chitinases [19] and another one is Δcps1 [20], which prevents the development of mature spherules by unknown mechanisms.

## References

[1] Edwards PQ, Palmer CE. Prevalence of sensitivity to coccidioidin, with special reference to specific and nonspecific reactions to coccidioidin and to histoplasmin. Dis Chest. 1957 1;31(1):35–60. PMID: 13384171. 13384171 10.1378/chest.31.1.35

[2] Benedict K, Jackson BB, Chiller T, et al Estimation of direct healthcare costs of fungal diseases in the United States. Clin Infect Dis. 2019;68(11):1791–1797. 30204844 10.1093/cid/ciy776 PMC6409199

[3] Martinez R New trends in paracoccidioides epidemiology. J Fungi. 2017;3(1). doi:10.3390/jof3010001. PMC5715958 29371520

[4] Sepulveda VE, Marquez R, Turissini DA, et al Genome sequences reveal cryptic speciation in the human pathogen *Histoplasma capsulatum*. MBio. 2017;5;8(6):pii: e01339-17 PMID:29208741/. doi:10.1128/mBio.01339-17. PMC5717386 29208741

[5] Fisher MC, Koenig GL, White TJ, et al Molecular and phenotypic description of *Coccidioides posadasii* sp. nov., previously recognized as the non-California population of *Coccidioides immitis*. Mycologia. 2002;94(1):73–84. PMID:21156479. 21156479

[6] Turissini DA, Gomez OM, Teixeira MM, et al Species boundaries in the human pathogen paracoccidioides. Fungal Genet Biol. 2017;106:9–25. Epub 2017 Jun 9. PMID:28602831. 28602831 10.1016/j.fgb.2017.05.007 PMC8335726

[7] McTaggert LR, Brown EM, Richardson SE Phylogeographic analysis of blastomyces dermatitidis and blastomyces gilchristii reveals an association with North American freshwater drainage basins. PLoS One. 2016;11(7):e0159396. 27428521 10.1371/journal.pone.0159396 PMC4948877

[8] Alvarez M, Casadevall A Phagosome extrusion and host-cell survival after Cryptococcus neoformans phagocytosis by macrophages. Curr Biol. 2006;16(21):2161–2165. PMID: 17084702. 17084702 10.1016/j.cub.2006.09.061

[9] Beyhan S, Sil A Sensing the heat and the host: virulence determinants of *Histoplasma capsulatum*. Virulence. 2019;10(01):793–800. 10.1080/21505594.2019.1663596 PMC6768255 31560240

[10] McBride JA, Gauthier GM, Klein BS Turning on virulence: mechanisms that underpin the morphologic transition and pathogenicity of *Blastomyces*. Virulence. 2019;10(01):801–809. PMID: 29532714. doi:10.1080/21505594.2018.1449506. PMC6779398 29532714

[11] Kirkland TN, Fierer J Coccidioides immitis and posadasii; A review of their biology, genomics, pathogenesis, and host immunity. Virulence. 2018;9(1):1426–1435. Review. PMID:30179067. 30179067 10.1080/21505594.2018.1509667 PMC6141143

[12] Kirkland TN Genetic differences between Coccidioides spp. and closely related nonpathogenic onygenales. bioRxiv. 2019. doi:10.1101/413906.

[13] Salzman SH, Smith RL, Aranda CP Histoplasmosis in patients at risk for the acquired immunodeficiency syndrome in a nonendemic setting. Chest. 1998;93(5):916–921. 10.1378/chest.93.5.916 3359846

[14] Wang H, LeBert V, Hung CY, et al C-type lectin receptors differentially induce Th17 cells and vaccine immunity to the endemic mycosis of North America. J Immunol. 2014;192(3):1107–1119. 24391211 10.4049/jimmunol.1302314 PMC3910401

[15] Calich VLG, Mammoni RL, Loures FV Regulatory T cells in Paracoccidioidomycosis. Virulence. 2019;10(01):810–821. PMID: 30067137. doi:10.1080/21505594.2018.1483674. PMC6779406 30067137

[16] Casadevall A, Coehlo C, Cordero RJB, et al The capsule of Cryptococcus neoformans. Virulence. 2019;10(01):822–831. PMID:29436899. doi:10.1080/21505594.2018.1431087. PMC6779390 29436899

[17] Nuῆez G, Sakamoto K, Soares MP Innate nutritional immunity. J Immunol. 2018;201(1):11–18. 29914937 10.4049/jimmunol.1800325 PMC6028930

[18] Wise HZ, Hung CY, Whiston E, et al Extracellular ammonia at sites of pulmonary infection with Coccidioides posadasii contributes to severity of the respiratory disease. Microb Pathog. 2013;59–60:19–28. 10.1016/j.micpath.2013.04.003 PMC3656146 23583291

[19] Xue J, Chen X, Selby D, et al A genetically engineered live attenuated vaccine of Coccidioides posadasii protects BALB/c mice against coccidioidomycosis. Infect Immun. 2009;77(8):3196–3208. 19487479 10.1128/IAI.00459-09 PMC2715678

[20] Narra HP, Shubitz LE, Mandel MA, et al A coccidioides posadasii CPS1 deletion mutant is avirulent and protects mice from lethal infection. Infect Immun. 2016;84:3007–3016. PMCID: PMC5038059 PMID: 27481239. 27481239 10.1128/IAI.00633-16 PMC5038059

